# Ultraprocessed Food Consumption and Behavioral Outcomes in Canadian Children

**DOI:** 10.1001/jamanetworkopen.2026.0434

**Published:** 2026-03-03

**Authors:** Meaghan E. Kavanagh, Zheng Hao Chen, Sukhpreet K. Tamana, Theo J. Moraes, Elinor Simons, Stuart E. Turvey, Padmaja Subbarao, Piushkumar J. Mandhane, Kozeta Miliku

**Affiliations:** 1 Department of Nutritional Sciences, Temerty Faculty of Medicine, University of Toronto, Toronto, Ontario, Canada; 2 Department of Nutrition, Harvard T.H. Chan School of Public Health, Boston, Massachusetts; 3 Department of Pediatrics, Faculty of Medicine, University of British Columbia, Vancouver, British Columbia, Canada; 4 Department of Paediatrics, Temerty Faculty of Medicine, University of Toronto, Toronto, Ontario, Canada; 5 Department of Translational Medicine, The Hospital for Sick Children, Toronto, Ontario, Canada; 6 Section of Allergy and Clinical Immunology, Department of Pediatrics and Child Health, Max Rady College of Medicine, University of Manitoba, Winnipeg, Manitoba, Canada; 7 Division of Allergy and Immunology, Department of Pediatrics, Faculty of Medicine, University of British Columbia, Vancouver, British Columbia, Canada; 8 Department of Physiology, Temerty Faculty of Medicine, University of Toronto, Toronto, Ontario, Canada; 9 Faculty of Health Sciences, McMaster University, Hamilton, Ontario, Canada; 10 Department of Pediatrics, Faculty of Medicine & Dentistry, University of Alberta, Edmonton, Alberta, Canada; 11 Faculty of Medicine, University College Sedaya International, Kuala Lumpur, Malaysia

## Abstract

**Importance:**

Ultraprocessed foods (UPF) contribute to nearly half of energy intake among preschool-aged children in Canada, yet their impact on behavioral and emotional functioning remains underexplored.

**Objective:**

To examine the associations between UPF intake at age 3 years and behavioral outcomes at age 5 years.

**Design, Setting, and Participants:**

This cohort study included children with dietary and behavioral data from September 2011 to April 2018 in the CHILD Cohort Study, a prospective Canadian pregnancy cohort. Data analysis was done between February to July 2025.

**Exposure:**

UPF intake at age 3 years was assessed by a 112-item food frequency questionnaire and categorized using the NOVA system.

**Main Outcomes and Measures:**

Behavior was measured using the validated Child Behavior Checklist (CBCL; range 0-100; higher scores indicating more adverse symptoms). UPF intake measured as a continuous percentage of energy was examined using multivariable-adjusted linear regression models accounting for maternal diet, birth factors, infant feeding, and sociodemographic and early-childhood characteristics. A multivariable-adjusted substitution model estimated the association of statistically replacing 10% energy from UPF with minimally processed foods (MPF) among all children.

**Results:**

Among 2077 participants, 1092 (52.6%) were male; 1376 children (66.2%) were White, 480 children (23.1%) were multiracial, and 221 children (10.7%) were identified as another ethnic group. At age 3 years, UPF contributed a mean (SD) of 45.5% (11.6%) of total energy intake. At age 5 years, the mean (SD) CBCL scores were 44.6 (9.1) for internalizing, 39.6 (9.4) for externalizing, and 41.2 (9.0) for total behavior. Each 10% increase in energy from UPF was associated with higher CBCL internalizing (β = 0.81 [95% CI, 0.43 to 1.19]), externalizing (β = 0.47 [95% CI, 0.08 to 0.87]), and total (β = 0.64 [95% CI, 0.27 to 1.01]) scores. Substitution of 10% energy from UPF with MPF was associated with lower internalizing (β = –0.91 [95% CI, –1.33 to –0.49]), externalizing (β = –0.49 [95% CI, –0.93 to –0.06]), and total (β = –0.70 [95% CI, –1.12 to –0.29]) scores.

**Conclusions and Relevance:**

In this cohort study of preschoolers in Canada, higher UPF intake was associated with adverse behavioral and emotional symptoms by age 5 years. These findings suggest that replacing UPF with MPF during the preschool years may support healthier behavioral development, with potential benefits for long-term mental health. These findings also support ongoing policy actions that promote MPF and underscore the need for early-life dietary interventions.

## Key Points

**Question:**

Are ultraprocessed foods (UPF) associated with behavioral and emotional functioning among preschool children?

**Findings:**

In this cohort study of 2077 Canadian children, higher UPF intake at age 3 years was associated with adverse behavioral and emotional symptoms at age 5 years. Modeling the substitution of a portion of UPF with minimally processed foods was associated with better behavioral and emotional symptoms.

**Meaning:**

These findings suggest that UPF consumption in early childhood may adversely influence behavioral and emotional development, and that ongoing public strategies promoting minimally processed foods in place of UPF could help support children’s development.

## Teaser

This cohort study examines the associations between intake of ultraprocessed foods at age 3 years and behavioral outcomes at age 5 years among children in Canada.

## Introduction

Early childhood is a critical period for behavior and emotional development, with long-term implications for psychosocial functioning and health trajectories.^1^ Adverse behavior symptoms that emerge during this time, including internalizing symptoms (eg, anxiety, withdrawal) and externalizing symptoms (eg, aggression, hyperactivity), are common pediatric concerns and are increasingly recognized as early indicators of later mental health.^2,3^ Globally, mental health disorders (eg, major depression, anxiety disorders) account for 32.4% of years lived with disability and 13.0% of disability-adjusted life years,^4^ and recent data suggest rising prevalence in children.^5^

Diet is a modifiable risk factor that may influence behavioral development, yet most of existing research has focused on nutrient-specific associations.^6,7,8^ Ultraprocessed foods (UPF) now dominate the modern food supply, displacing traditional whole and minimally processed foods (MPF).^9^ In Canada, UPF contributed to nearly half (48%) of daily energy intake among preschool-aged children.^10,11^ These foods are industrially formulated with additives not commonly used in home cooking to maximize palatability, shelf-life, and profit.^12^ Although UPF offer convenience, affordability, and microbiological safety, they are typically energy-dense and high in sugar, saturated fat, and sodium, while low in fiber and essential nutrients.^13^

Mounting evidence links UPF to adverse health outcomes,^14^ including elevated risk of obesity and cardiometabolic diseases in both adults and children.^11,15,16,17^ Emerging research also suggests associations between high UPF intake and adverse behavior and mental health outcomes, particularly in adults and adolescents.^18,19,20,21,22,23,24^ For example, a cross-sectional study of Spanish adolescents reported that higher UPF intake was associated with higher internalizing (eg, anxiety, withdrawal) and externalizing (eg, disruptive behavior, challenges with conduct) symptoms.^18^

Despite these findings, the influence of UPF intake on behavioral development during early childhood remains unexplored,^24,25^ although limited evidence examining other dietary characteristics suggests associations with hyperactivity and attention-deficit/hyperactivity disorder–related behaviors.^26,27,28^ Recent calls to action emphasize the need to integrate psychosocial outcomes more often in nutrition research, acknowledging their bidirectional associations with diet and other behaviors.^29^ Given that UPF contribute almost half of all calories consumed in Canadian preschool children,^10^ understanding their influence on early-life behavioral development is crucial. Several existing policies encourage limiting highly processed foods, including Canada’s Food Guide,^30^ and federal front-of-pack labeling and marketing restrictions.^31,32^ Clinical guidance also supports whole-food dietary patterns for mental and behavioral health, including recommendations from the American Psychiatric Association.^33^ Therefore, in one of the largest population-based Canadian cohorts, the CHILD Cohort Study, we aimed to examine the associations between preschool UPF intake and behavioral outcomes.

## Methods

This cohort study was conducted as part of the CHILD Cohort Study, which was approved by the research ethics boards at McMaster University and Universities of Manitoba, Alberta, and British Columbia and the Hospital for Sick Children. This study was approved by the human research ethics boards at University of Toronto. Informed consent was obtained from caregivers. We report this study in accordance with the Strengthening the Reporting of Observational Studies in Epidemiology (STROBE) reporting guidelines for cohort studies.

### Study Design and Population

The CHILD Cohort Study is a longitudinal, multicenter, population-based pregnancy cohort study that has been described in detail elsewhere.^34,35^ A total of 3621 pregnant women were recruited between 2008 and 2012 from 4 study sites across central to western Canada, including Vancouver, Edmonton, Manitoba (Winnipeg, Morden/Winkler), and Toronto. The cohort is broadly representative of the sociodemographic characteristics of these regions, with distributions of income, education, and ethnicity comparable to national data, although participating mothers tended to have slightly higher educational background.^34,35^ Participants remained eligible if they were from singleton births, gestational age of at least 34 weeks and 4 days, and had no congenital abnormalities (3454 individuals). For this study, we excluded participants with a diagnosis of Trisomy 21 (6 individuals) and who withdrew before the 3-year visit (222 individuals), with missing dietary data at age 3 years (792 individuals), missing more than 12 questions from the food frequency questionnaire (FFQ) (5 individuals), or had outliers for energy intake identified using the Tukey far-out fence method (25 individuals) (eFigure 1 in Supplement 1).^36^ Among 2404 children with available dietary data at age 3 years, 327 with no behavioral data collected at age 5 years were excluded. Our final study population included 2077 participants with valid dietary data at the 3-year visit and behavioral outcome data at the 5-year visit (September 2011 to April 2018) (eFigure 1 in Supplement 1).

### Preschool Dietary Intake of UPF

UPF at the 3-year visit were assessed according to the NOVA classification system,^37^ from a 112-item semiquantitative FFQ completed by caregivers. The FFQ was validated among a subset of the Family Atherosclerosis Monitoring in Early Life study.^38,39^ We have previously described in detail how UPF were derived^11^ and have provided further details in eMethods 1 in Supplement 1. Briefly, all FFQ items were mapped following past examples^40^ to the 4 NOVA groups: (1) unprocessed and MPF; (2) processed culinary ingredients; (3) processed foods; and (4) UPF.^37^ NOVA groups were calculated as percentage energy contributed, with energy intake from each group being divided by the total daily energy intake, multiplied by 100. UPF were further categorized into 8 mutually exclusive subgroups (ie, sweets and desserts; breads and cereals; animal-based products; ready-to-eat or ready-to-heat mixed dishes; artificially and sugar-sweetened beverages; sauces, spreads, and condiments; savory snacks; and plant-based alternatives), defined elsewhere.^41^

### Child Behavior Outcomes

At the 5-year visit, behavioral and emotional outcomes were assessed using the validated preschool version (ages 1.5-5 years) of the Child Behavior Checklist (CBCL) caregiver-reported instrument. The CBCL includes 99 statements describing a range of behavioral and emotional symptoms.^42^ Caregivers or parents are asked to rate each statement on a 3-point scale (0-2), with 0 being not true of the child, 1 being somewhat or sometimes true, and 2 being very true or often true. As described elsewhere,^43,44^ the CBCL is standardized to a normal population (adjusted for age) and represented by a T-score with mean (SD) of 50 (10), with a higher score indicating more caregiver-reported behavior problems (range, 0-100).^45^ The total behavior scale includes questions from the internalizing scale, which captures inward-focused behavior (eg, anxiety, fearfulness, and depression), and externalizing scale, which captures outward-directed behavior (eg, aggression, hyperactivity), in addition to sleep problems and other problems.

### Covariates

Confounders were chosen based on literature and using a directed acyclic graph.^46^ These included prenatal stress, assessed using the Perceived Stress Scale (range, 0-40; higher scores indicating more stress),^47^ maternal UPF during pregnancy (percentage of kilocalories), maternal education (postsecondary degree vs none), maternal marital status (married, single, or divorced), child sex (female or male), caregiver-reported child ethnicity (White, multiracial, or other), gestational age at birth (weeks), exclusive breastfeeding at 6 months (yes vs no), older siblings (yes vs no), household income (<$50 000, $50 000-$99 999, ≥$100 000, or prefer not to not say [approximately <US $36 191, $36 191-$72 381, and US $72 382, respectively]), study site (Vancouver, Edmonton, Manitoba, or Toronto), energy intake at 3 years (kilocalories per day), season at 3-year study visit (spring, summer, winter, autumn), attendance of childcare settings at 3 years (yes vs no), physical activity at 5 years (hours per week), and body mass index *z* score (World Health Organization reference standards)^48^ at 5 years. We did not adjust for age, as the CBCL is standardized across age groups.^43^ Ethnicity was caregiver-reported using a questionnaire with response options Caucasian White, Black, East Asian, First Nations, Hispanic, Middle Eastern, mixed, South Asian, South-East Asian, and other. For analysis, we presented Caucasian White as *White*, mixed as *multiracial*, and grouped all remaining categories as *other* due to small numbers following contemporary race and ethnicity reporting guidelines.^49^

### Statistical Analysis

Continuous normally distributed variables were expressed as mean and SD, continuous non–normally distributed variables expressed as median and IQR, and categorical variables expressed as number and percentage. We performed nonresponse analyses to compare characteristics of participants included in our study with dietary exposures at age 3 years and CBCL outcome at age 5 years (2077 participants) and participants excluded due to missing child behavioral data at the 5-year visit (327 participants). We used multivariable-adjusted linear regression analysis accounting for maternal, infancy, and childhood confounders to examine the associations of UPF energy percentage with child behavior outcomes. In sensitivity analyses, we accounted for (1) nutrients of concern related to UPF intake (total sugar, sodium, and saturated fat), (2) the change in UPF intake between ages 3 and 5 years, and (3) child screen time at age 5 years (hours per day). To assess whether associations differed by child sex, we evaluated the statistical interaction by including the product term with UPF in the main model; there was no evidence of effect modification (all interaction *P* values >.20).

Furthermore, we statistically modeled substituting 10% of energy contributed by UPF for the equivalent energy contribution from MPF. Both variables were introduced simultaneously into the model, and the differences in the β coefficients, variances, and covariance were used to estimate the β and 95% CIs for the isocaloric substitution.^50^ This approach is an established method to assess health impacts of dietary shifts,^50^ and is particularly relevant for policy contexts where reductions in one food imply increases in another under isocaloric conditions.^51^ UPF subgroups were also examined to explore whether specific types of UPF were differentially associated with behavioral outcomes.^41^

To limit potential bias associated with missing data (ranging from 0.3%-9.1%), missing values of covariates among 2077 participants with exposure and outcome data were imputed (n = 5) using fully conditional specification (predictive mean matching for continuous variables, logistic regression for binary variables, random forest for multilevel categorical variables), assuming no monotone missing pattern.^52^ We reported pooled effect estimates after multiple imputations.^53^ Participant characteristics before and after imputations are shown in eTable 1 in Supplement 1, and full imputation specifications are provided in eMethods 2 in Supplement 1. A complete-case analysis was run among 1492 participants with no missing covariate data to assess strength of the association.

All statistical analyses were performed using R software version 12.0 (R Project for Statistical Computing). *P* values were 2-sided, and *P* < .05 was prespecified to be considered significant. However, to align with American Statistical Association recommendations, associated estimates and uncertainties were interpreted in context rather than relying on a strict *P* value threshold.^54^ Data analysis was done between February 1 and July 30, 2025.

## Results

### Participant Characteristics

Among 2077 participants, 1092 (52.6%) were male; 1376 children (66.2%) identified as White, 480 children (23.1%) as multiracial, and 221 children (10.7%) as another ethnicity; 1186 children (57.1%) were from households with an income of $100 000 CAD or greater (Table). Descriptive characteristics of 327 participants excluded due to missing child behavioral data are shown in eTable 2 in Supplement 1. Participants included in the analysis were more likely to have mothers with postsecondary education (79.0% vs 66.3%) and household income of $100 000 CAD or greater (52.3% vs 34.9%) compared with those not included in the analyses. No differences were observed in maternal stress during pregnancy or maternal and child UPF intake (eTable 2 in Supplement 1). At the 3-year visit, energy intake as median (IQR) was 1488.6 (1211.9-1817.2) kcals/d, where UPF accounted for a mean (SD) of 45.5% (11.6%) of energy intake and MPF accounted for 37.9% (11.1%) of energy intake (Table).

**Table.** Study Population Characteristics in the CHILD Cohort Study

Characteristic^a^	Participants, No. (%) (N = 2077)
Family level	
Maternal education (postsecondary degree)	1674 (80.6)
Maternal stress during pregnancy, mean (SD)	12.1 (6.1)
Maternal marital status	
Married	1986 (95.6)
Single	79 (3.8)
Divorced	12 (0.6)
Maternal energy intake contributed from UPF, mean (SD), %	46.6 (10.6)
Annual household income, CAD $^b^	
<$50 000	187 (9.0)
$50 000-$99 999	582 (28.0)
≥$100 000	1186 (57.1)
Prefer not to say	122 (5.9)
Older siblings (yes vs no)	955 (46.0)
Child level	
Sex	
Female	985 (47.4)
Male	1092 (52.6)
Ethnicity^c^	
White	1376 (66.2)
Multiracial	480 (23.1)
Other	221 (10.7)
Gestational age, median (IQR), d	278.0 (272.0-284.0)
Exclusive breastfeeding at 6 mo (yes vs no)	385 (18.5)
Daily energy intake at age 3 y, median (IQR), kcal/d	1488.6 (1211.9-1817.2)
Energy contribution of UPF at age 3 y, mean (SD), %	45.5 (11.6)
Energy contribution of MPF at age 3 y, mean (SD), %	37.9 (11.1)
Childcare attendance at age 3 y (yes vs no)	1218 (58.6)
Screen time at age 5 y, median (IQR), h/d	1.3 (1.0-2.1)
Physical activity at age 5 y, median (IQR), h/wk	2.0 (1.0-3.0)
BMI *z* score at age 5 y , mean (SD)	0.3 (1.0)
CBCL *t*-scores at age 5 y, mean (SD)	
Internalizing	44.6 (9.1)
Externalizing	39.6 (9.4)
Total	41.2 (9.0)

Abbreviations: BMI, body mass index (calculated as weight in kilograms divided by height in meters squared, using World Health Organization growth standards); CBCL, Child Behavior Checklist; MPF, minimally processed food; UPF, ultraprocessed food.

^a^ Characteristics are the pooled values after multiple imputations (5 imputation).

^b^ Approximately <US $36 191, $36 191-$72 381, and US $72 382, respectively.

^c^ Ethnicity was caregiver-reported. Questionnaire response options included: Caucasian White, Black, East Asian, First Nations, Hispanic, Middle Eastern, mixed, South Asian, South-East Asian, and other. For analysis, we presented Caucasian White as White, mixed as multiracial, and grouped all remaining categories as other due to small numbers.

**Figure 1.**
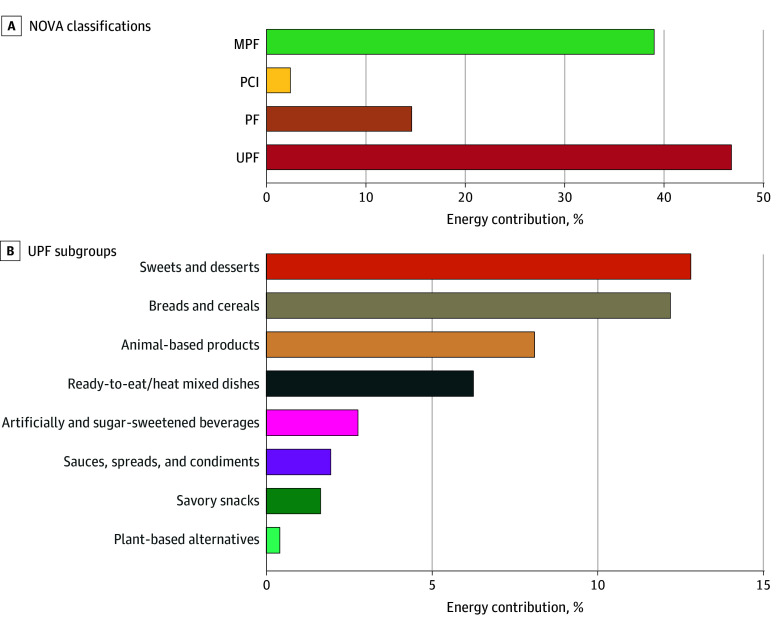
Bar Graphs of Daily Energy Intake Contributed by Each NOVA Group and Ultraprocessed Foods (UPF) Subgroups in the CHILD Cohort Study (N = 2077) A, Bar graph of energy intake contributed by each NOVA Classification System group. MPF indicates minimally processed foods; PCI, processed culinary ingredients; PF, processed foods. B, Bar graph of UPF subgroups that make up the total energy contributed by UPF items. The assumptions made during the NOVA classification mapping process are provided in eMethods 1 in Supplement 1.

Figure 1A presents dietary shares of energy intake contributed by each NOVA group. The main UPF subgroups contributing to energy were sweets and desserts (12.5%), breads and cereals (11.9%), animal-based products (7.9%), and ready-to-eat or ready-to-heat mixed dishes (6.1%) (Figure 1B). At the 5-year visit, the mean (SD) for internalizing, externalizing, and total CBCL scores were 44.6 (9.1), 39.6 (9.4), and 41.2 (9.0), respectively.

### Child UPF Intake and Behavior Scores

In the crude analyses, every 10% energy contribution from UPF was associated with higher scores across all CBCL domains at the 5-year visit (internalizing: β = 1.02 [95% CI, 0.68-1.35]; externalizing: β = 0.99 [95% CI, 0.65-1.34]; total score: β = 1.05 [95% CI, 0.72-1.38]) (eFigure 2 in Supplement 1). These associations were slightly attenuated but remained significant in the multivariable-adjusted regression analyses, where higher UPF intake was associated with higher scores across all CBCL domains (internalizing: β = 0.81 [95% CI, 0.43-1.19]; externalizing: β = 0.47 [95% CI, 0.08-0.87]; total score: β = 0.64 [95% CI, 0.27-1.01]). These associations remained consistent across the CBCL outcomes even after accounting for nutrients of concern (eg, saturated fat, sodium, sugar) and the change in UPF intake between ages 3 and 5 years (eFigure 2 in Supplement 1). Additionally, in the complete-case analysis of 1492 participants, associations remained significant and consistent with the main multivariable adjusted analysis (eFigure 3 in Supplement 1).

### Substitution of UPF for MPF

**Figure 2.**
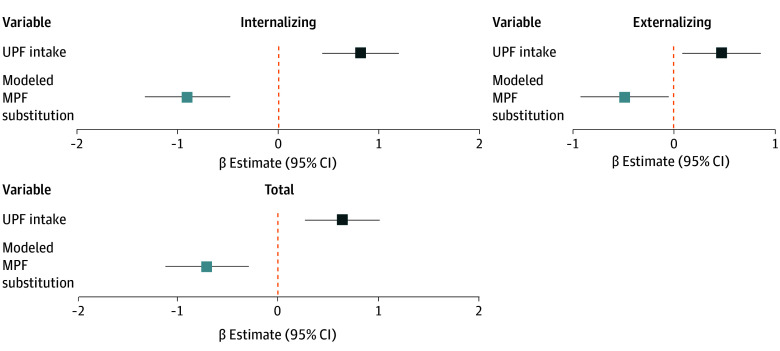
Forest Plots of Associations of Ultraprocessed Foods (UPF) and Substitution of UPF With Minimally Processed Foods (MPF) and Child Behavioral Checklist Scores in the CHILD Cohort Study (N = 2077) Charts depict linear regression analyses between every 10% increase in UPF intake and Child Behavioral Checklist T-scores (dark blue), and modeled substitution of 10% of energy from UPF for MPF, where β estimates represent the expected change in CBCL scores (light blue). The models are adjusted for prenatal stress, maternal UPF, maternal education, maternal marital status, child sex, child ethnicity, gestational age, exclusive breastfeeding at 6 months, older siblings, household income, study site, energy intake at age 3 years, season at 3-year study visit, attendance of childcare settings at age 3 years, physical activity at age 5 years, and body mass index *z* score at age 5 years.

Figure 2 presents the estimated change in CBCL scores associated with statistically substituting 10% of energy from UPF with 10% of energy from MPF, while holding total energy intake and all other covariates constant. Substitution of UPF with MPF was associated with lower scores across all domains (internalizing: β = −0.91 [95% CI, −1.33 to −0.49]; externalizing: β = −0.49 [95% CI, −0.93 to −0.06]; total score: β = −0.70 [95% CI, −1.12 to −0.29]).

### UPF Subgroups at Age 3 Years and Child CBCL Scores

**Figure 3.**
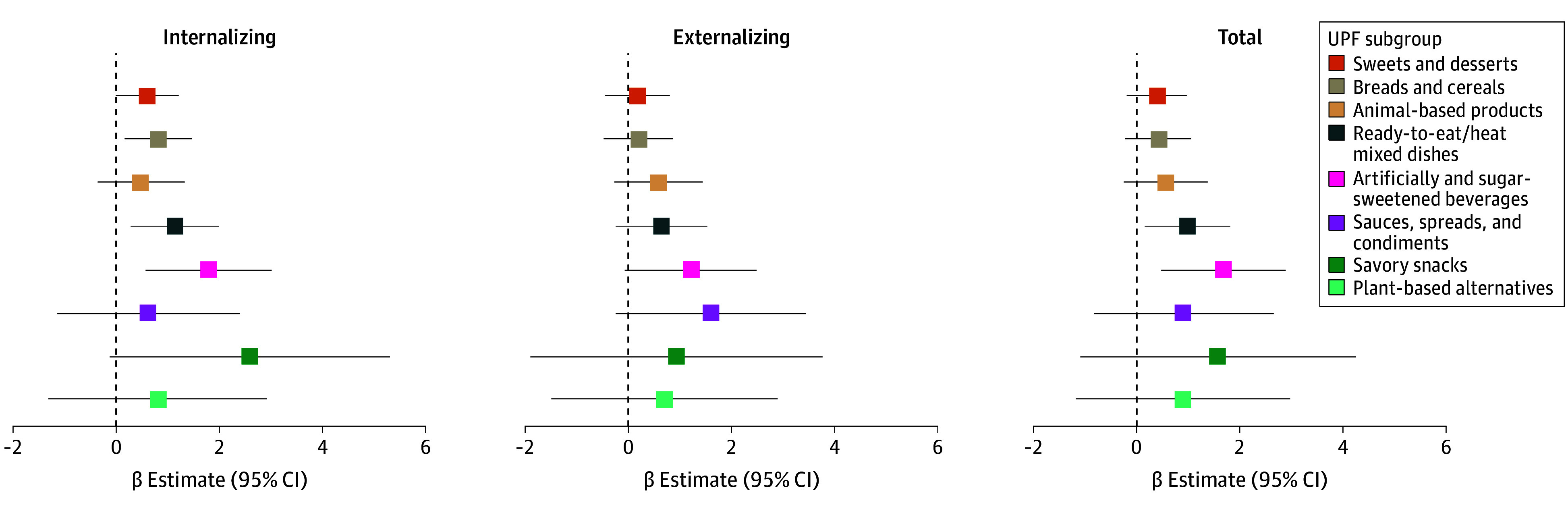
Forest Plots of Association Between Group-Specific Ultraprocessed Foods (UPF Intakes and Child Behavioral Checklist Scores in the CHILD Cohort Study (N = 2077) Charts depict multivariable-adjusted regression analyses. All UPF groups were simultaneously included in the model as distinct covariables, adjusting for prenatal stress, maternal UPF, maternal education, maternal marital status, child sex, child ethnicity, gestational age, exclusive breastfeeding at 6 months, older siblings, household income, study site, energy intake at age 3 years, season of 3-year study visit, attendance of childcare settings at age 3 years, physical activity at age 5 years, and body mass index *z* score at age 5 years.

Figure 3 presents the multivariable-adjusted regression analyses by UPF subgroups and CBCL scores. Higher intake of artificially and sugar-sweetened beverages was associated with a higher internalizing score (β = 1.76 [95% CI, 0.53 to 2.98]) and total behavior score (β = 1.65 [95% CI, 0.45 to 2.85]) but not externalizing (β = 1.22 [95% CI, –0.05 to 2.50]). There was no strong evidence of associations across other UPF subgroups, except that higher intakes of breads and cereals and ready-to-eat or ready-to-heat mixed dishes were associated with higher internalizing scores (breads and cereals : β = 0.79 [95% CI, 0.14 to 1.44]; ready-to-eat or ready-to-heat mixed dishes: β = 1.11 [95% CI, 0.27 to 1.95]).

## Discussion

In this prospective Canadian cohort study of young children, higher UPF intake at age 3 years was associated with adverse behavioral and emotional symptoms at age 5 years. Modeling substitution of UPF for MPF was associated with lower CBCL scores, suggesting that efforts encouraging MPF in place of UPF may help to support child behavioral, emotional, and mental health development.

To our knowledge, this is the first study to examine UPF consumption and standardized behavioral assessments in children using detailed, prospective data. In this study, we report novel findings that substitution of UPF for MPF in terms of calorie contribution was associated with lower CBCL scores, highlighting a potential dietary modification target. Our results align with prior work from the UK in which junk food intake, defined as high-fat processed foods (burgers, coated poultry, crisps, and chocolate), at age 4.5 years was associated with higher hyperactivity at age 7 years.^26^ Our study builds on this work by assessing multiple behavioral domains using the CBCL and adjusting for a wide range of prenatal and early-life confounders. When UPF were examined by subgroup, higher intakes of artificially and sugar-sweetened beverages were associated with higher internalizing and total behavior scores, and higher intakes of breads and cereals and ready-to-eat or ready-to-heat mixed dishes were associated with higher internalizing scores. The heterogeneity observed across UPF subgroups is consistent with other cohort studies,^41,55^ which suggests that not all UPF carry the same health risks, supporting the value of subgroup analyses to identify potentially more harmful product categories.

Our findings also support broader literature linking healthier dietary patterns with better psychosocial outcomes. Studies in children and adolescents have observed associations between higher diet quality with fewer depressive symptoms,^6^ while certain dietary components, such as food additives, refined sugars, and fatty acids, have been investigated for potential links to hyperactivity disorders, although evidence remains limited.^7^

In adults, dietary interventions have improved depressive symptoms.^56^ Notably, a randomized clinical trial in young males with clinical depression found improvements with a Mediterranean diet intervention.^57^ Together, these findings strengthen the case for a causal link between diet quality and mental health. However, robust experimental studies in young children are needed to confirm these associations and explore underlying mechanisms.

The mechanisms linking UPF consumption with early childhood behavioral and emotional development remain poorly understood, but several biological pathways have been proposed. UPF are generally low in fiber and micronutrient density and high in saturated fat, sugars, sodium, and energy density. These nutrients have been implicated in neurobiological pathways, with saturated fats promoting neuroinflammation and altered gut-brain signaling,^58^ excess sodium linked to heightened stress responsivity and neuroinflammatory processes,^59^ and high sugar intake associated with emotional dysregulation and depressive symptoms.^60^ These pathways are biologically plausible and supported by emerging literature but were not directly measured in our analysis. In our main models, we adjusted for total energy intake, and in a sensitivity analysis, we further accounted for nutrients of concern, calculated from a nutrient matrix. Associations persisted after adjustment for sugar, sodium, and saturated fat at age 3 years, suggesting that nutrients of concern do not explain the observed associations.

Our subgroup analyses suggest possible nutrient-displacement, whereby energy from UPF (eg, artificially and sugar-sweetened beverages) replaces nutrient-dense foods.^61^ Such displacements may contribute to deficiencies in vitamin D, zinc, iron, folate, vitamin B12, and omega-3 fatty acids, which are critical for neurotransmitter function. Deficits in these nutrients have been associated with depression-related outcomes in children and adolescents,^62,63,64^ leading to nutritional medicine becoming mainstream in psychiatry.^65^ Although most subgroup estimates included the null, the consistent direction of the associations across all UPF subgroups suggests that mechanisms beyond nutrient composition may be relevant, including exposure to endocrine disrupting chemicals from UPF packaging materials containing phthalates and bisphenol.^66^ Given rapid developmental processes, young children may be particularly sensitive to such exposures.^67,68^

A separate consideration is the gut-brain axis.^69^ UPF have been suggested to influence the gut microbiome and immune and inflammatory responses,^70,71,72,73^ all potentially impacting brain function. Studies in infants and adults have shown that higher UPF intake or highly processed diets are associated with lower microbial diversity,^74^ impaired cytokine responses,^72^ and higher markers of systemic inflammation, including C-reactive protein.^70^ Understanding these mechanisms will be essential for informing policies and reformulation strategies aimed at mitigating the potential neurodevelopmental impacts of UPF.

Understanding the influence of dietary choices in early childhood is critical, as dietary patterns are established early in life and track into adulthood, and particularly as UPF contribute to nearly half (46%) of total caloric intake among Canadian children.^10^ Emotional and behavioral development during the preschool years is shaped by a complex interplay of exposures. Identifying modifiable early-life exposures, such as diet, is important, given that psychosocial risk indicators, including those captured by the CBCL, may predict later mental health outcomes.^3^

Our findings suggest that early-life UPF intake was associated with more behavioral and emotional symptoms at age 5 years. Although the effect sizes were modest, they are consistent with other cohorts that link early-life exposures, including diet, to CBCL outcomes, where small shifts (0.3-1.0 points) are associated with later behavioral trajectories.^2,3,26^ These associations emerged during the preschool years, a sensitive developmental period when early differences tend to persist.^75,76^ Given the high prevalence of UPF consumption, even modest associations may have meaningful population-level implications, particularly given links between mental and physical health.^77^ Early dietary habits are also associated with later cardiometabolic risk factors,^78^ underscoring preschool years as a key window for intervention.

Our study reinforces the need for strategies in preschool children, ranging from health care professional guidance for families, public health campaigns and childcare nutrition standards to reformulation of specific UPF categories, that promote MPF during critical periods of development. These priorities align with Canada’s existing national policies (eg, front-of-pack warning labels and restricting advertising directed at children for foods high in nutrients of concern)^31,32^ and with global recommendations, including the recent *Lancet* series on UPF promoting minimally processed diets,^79,80^ and with clinical guidance recommending whole-food dietary patterns to support mental health.^33^ Strengthening early-life nutrition strategies within these frameworks may offer additional benefits for children’s development.

### Strength and Limitations

Our study’s strengths include its prospective design, large and geographically diverse study population, and comprehensive data allowing adjustment for numerous covariates. Behavioral outcomes were measured using the validated CBCL, which is widely applied in pediatric research.^42^ We examined both total and subgroup-specific UPF intake to assess potential heterogeneity and used substitution models to estimate the potential impact of replacing UPF with MPF.

Our study also has limitations. Although NOVA is widely used, it may group nutritionally distinct foods and has shown divergent associations across UPF categories^41,55,81^; despite our subgroup analyses, compositional diversity remains a challenge. We did not determine whether higher UPF intake displaced nutrient-dense foods; therefore, potential nutrient-displacement pathways remain speculative. Although UPF are often lower in micronutrient density,^82^ particularly compared with MPF,^83^ micronutrient content varies across UPF categories, and some products are fortified. Future work should examine dietary patterns in greater detail to determine whether UPF consumption contributes to micronutrient insufficiencies relevant to behavioral and emotional development.

Additionally, dietary intake was assessed using FFQs, which are subject to recall and reporting bias^84^ and have inherent limitations for UPF classification. FFQs do not capture ingredient lists or whether foods were homemade or commercially produced; therefore, assumptions must be made when assigning NOVA categories. As in other cohort studies,^40^ we applied a conservative approach, which may bias estimates toward the null. Although misclassification is possible, our UPF estimates align with national intakes derived from 24-hour dietary recalls.^10^ As well, a recent concordance analysis reported acceptable agreement between a standard FFQ and the NOVA-specific FFQ.^85^ Future studies incorporating more detailed dietary ascertainment or barcode-based methods could further strengthen NOVA classification accuracy. As with any observational studies, causal inference is limited. Despite careful adjustment, residual confounding cannot be ruled out.

## Conclusions

In this prospective cohort study of preschoolers in Canada, higher UPF intake at age 3 years was associated with more behavioral and emotional problems at age 5 years. Although effect sizes were modest, such differences could be of significance at the population level. These findings reinforce existing strategies that promote minimally processed diets and highlight the importance of strengthening early-life nutrition policies to support healthy behavioral development and potentially improve later mental health.

## Notes

Supplement 1. **eMethods 1.** Food Frequency Questionnaire **eMethods 2.** Imputation Specifications **eFigure 1.** Flowchart of the study population **eFigure 2.** Sensitivity analyses of associations of ultraprocessed food intake and CBCL scores in the CHILD study (n=2077) **eFigure 3. **Multivariable-adjusted association of UPF intake at age three and CBCL scores at age five - complete case analysis (n=1492) **eTable 1.** Descriptive characteristics of the original and multiple imputed datasets (n=2077) in the CHILD Cohort Study **eTable 2.** Descriptive characteristics and non-response analysis of participants included in the analysis (n=2077) and participants excluded due to missing child behavioural checklist (CBCL) data (n = 327) in the CHILD Cohort Study
eReferences.

Supplement 2.
Data Sharing Statement
