# Supplementary material for: Ultraprocessed Food Consumption and Behavioral Outcomes in Canadian Children
Source: JAMA Netw Open. 2026 Mar 3;9(3):e260434. doi: 10.1001/jamanetworkopen.2026.0434 (PMC12958080; doi:10.1001/jamanetworkopen.2026.0434)
Supplement: Supplement 1. — eMethods 1. Food Frequency Questionnaire eMethods 2. Imputation Specifications eFigure 1. Flowchart of the study population eFigure 2. Sensitivity analyses of associations of ultraprocessed food intake and CBCL scores in the CHILD study (n=2077) eFigure 3. Multivariable-adjusted association of UPF intake at age three and CBCL scores at age five - complete case analysis (n=1492) eTable 1. Descriptive characteristics of the original and multiple imputed datasets (n=2077) in the CHILD Cohort Study eTable 2. Descriptive characteristics and non-response analysis of participants included in the analysis (n=2077) and participants excluded due to missing child behavioural checklist (CBCL) data (n = 327) in the CHILD Cohort Study eReferences. [file jamanetwopen-e260434-s001.pdf]

Supplemental Online Content

Kavanagh ME, Chen ZH, Tamana SK, et al. Ultraprocessed food consumption and behavioral outcomes in Canadian children. *JAMA Netw Open*. 2026;9(3):e260434. doi:10.1001/jamanetworkopen.2026.0434

eMethods 1. Food Frequency Questionnaire
eMethods 2. Imputation Specifications
eFigure 1. Flowchart of the study population
eFigure 2. Sensitivity analyses of associations of ultraprocessed food intake and CBCL scores in the CHILD study (n=2077)
eFigure 3. Multivariable-adjusted association of UPF intake at age three and CBCL scores at age five - complete case analysis (n=1492)
eTable 1. Descriptive characteristics of the original and multiple imputed datasets (n=2077) in the CHILD Cohort Study
eTable 2. Descriptive characteristics and non-response analysis of participants included in the analysis (n=2077) and participants excluded due to missing child behavioural checklist (CBCL) data (n = 327) in the CHILD Cohort Study
eReferences.

This supplemental material has been provided by the authors to give readers additional information about their work.

© 2026 Kavanagh ME et al. *JAMA Network Open*.

Supplementary Information (eMethods)

eMethod 1: Food Frequency Questionnaire

The FFQ items were mapped to the four NOVA groups with MPF (e.g., whole fruits, vegetables, eggs, rice), processed culinary ingredients (e.g., butter.), processed foods (e.g., tofu, peanut butter) and UPF (e.g., cold cereal, soda, cold cut deli meat slices). Because the FFQ did not ask about prepared/made at home, assumptions were made when mapping FFQ items. All food items were independently mapped by two researchers. Ambiguous items and/or conflicting food mappings were discussed with a third researcher to reach a consensus. For food items where it could fall under two or more categories based on processing (e.g. pizza can be group 3 if it is homemade, or group 4 if it is store brought or restaurant made) a conservative approach taken to classifying foods in the lesser processed group, following past work (Khandpur et al. 2021).[1] The FFQ assessed intake frequency in the past month, with each item having a 9-level scale to assess intake frequency ranging from “none” to “greater than 3 times per day”. Energy intake of each food item was estimated using a nutrient matrix created from the 2019-2020 Food and Nutrients Database for Dietary Studies from the US Department of Agriculture. The percentage of daily energy contributed from UPF intake was calculated by dividing the energy intake from UPF by the total daily energy intake of the whole diet, then multiplied by 100. To allow for standardization for modeling UPF exposure across studies 10-percentage-point increments in the % of total energy intake have been specifically recommended.[2]

eMethods 2: Imputation Specifications

The imputation dataset included all variables in the multivariable-adjusted model with missing values (e.g., gestational weeks, exclusive breastfeeding, physical activity, daily screen time), variables in the multivariable-adjusted model without missing values including exposure and outcomes (used as indicators; e.g., UPF intake, CBCL scores, child sex, study site, having older siblings, and dietary variables at age 3 years [energy intake, total sugars, sodium, total saturated fatty acids]), and other indicator variables that will help the prediction algorithm with imputation (e.g., total breastfeeding duration). No external bounds were imposed; plausibility was ensured through method-specific constraints (e.g., predictive mean matching draws from observed values).

© 2026 Kavanagh ME et al. *JAMA Network Open*.

FIGURES

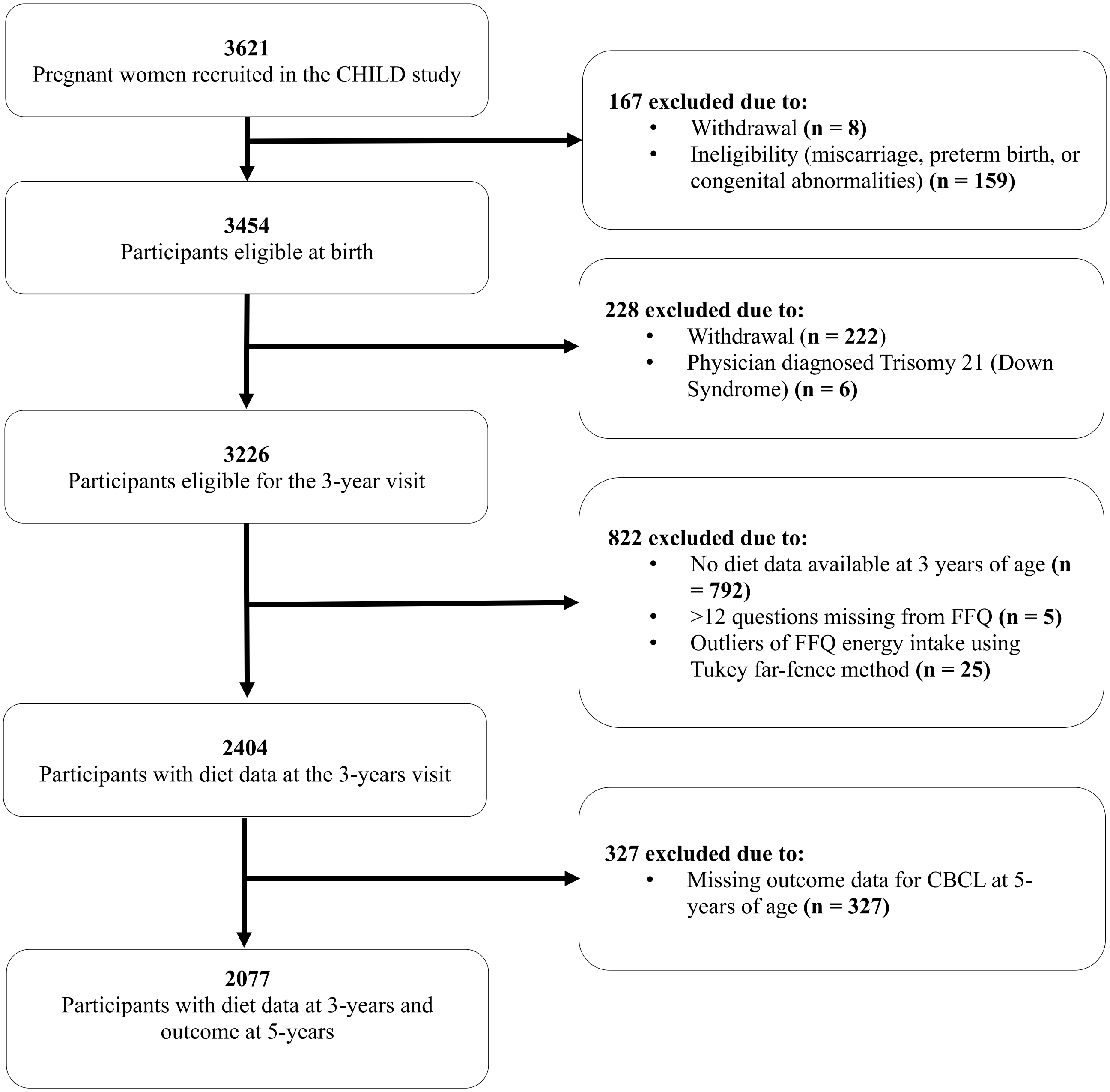

eFigure 1. Flowchart of the study population

© 2026 Kavanagh ME et al. *JAMA Network Open*.

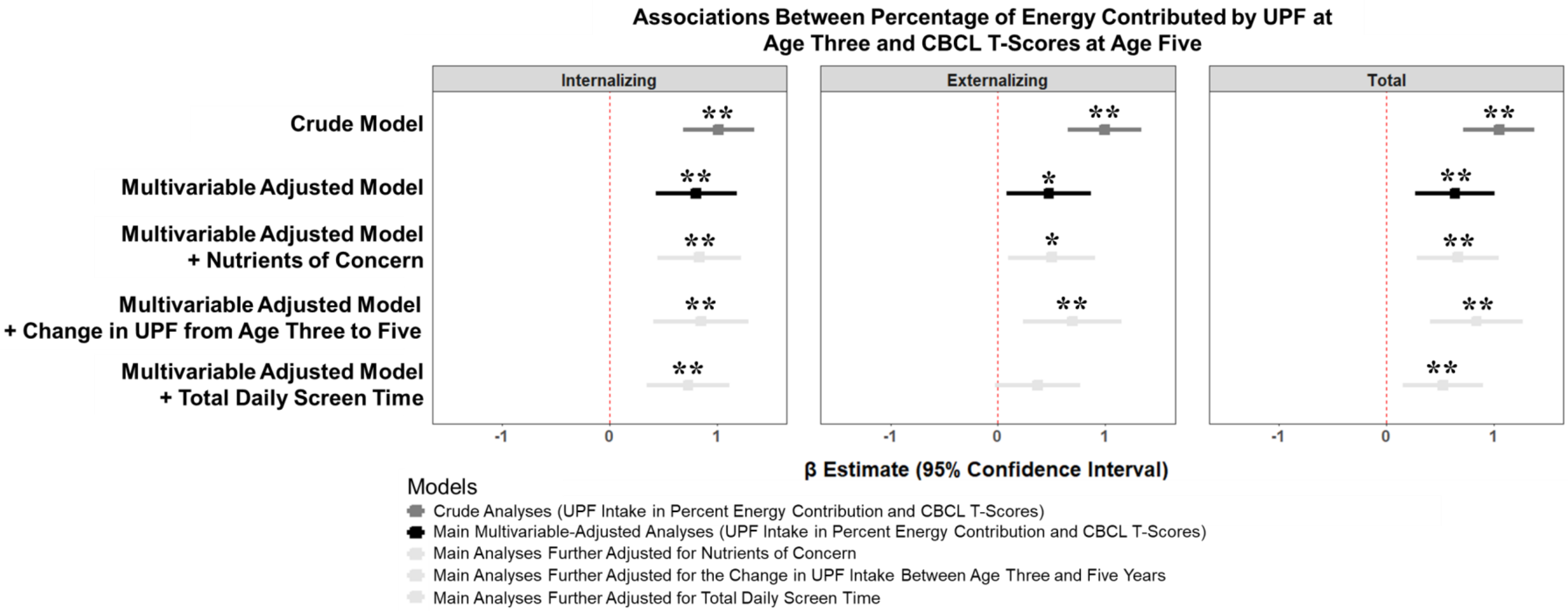

eFigure 2. Sensitivity analyses of associations of ultraprocessed food intake and CBCL scores in the CHILD study (n=2077)
Values are beta estimates with 95% confidence intervals from linear regression analyses of every 10% increase in energy intake from ultraprocessed food (UPF) intake at age three years and their associations with CBCL scores at age five years.

© 2026 Kavanagh ME et al. *JAMA Network Open*.

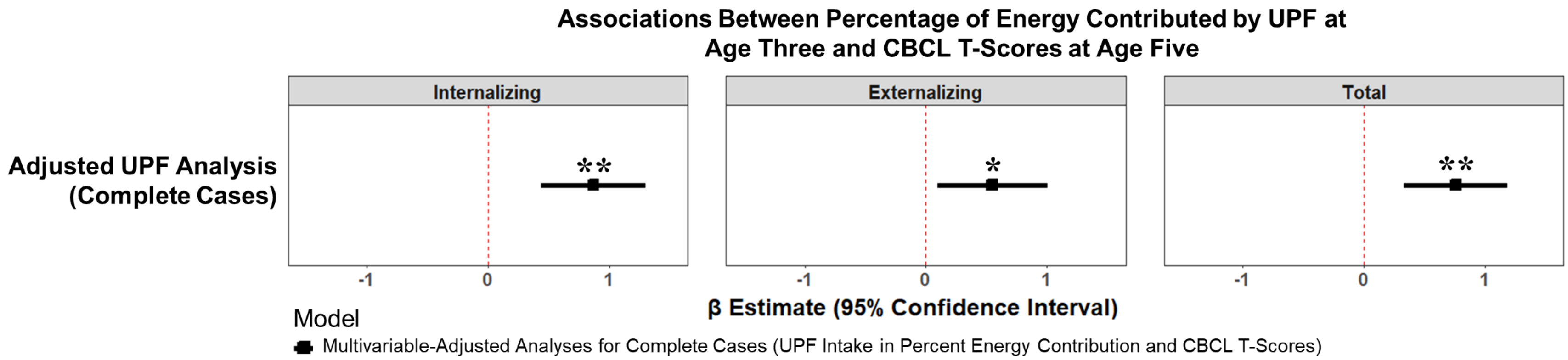

eFigure 3. Multivariable-adjusted association of UPF intake at age three and CBCL scores at age five - complete case analysis (n=1492)

Values are beta estimates with 95% confidence intervals from linear regression analyses of every 10% increase in energy intake from ultraprocessed food (UPF) intake at age three years and their associations with CBCL scores at age five years. Models are adjusted for prenatal stress, maternal UPF (percent of total daily kcal), maternal education (post-secondary degree vs. none), maternal marital status (married; single; divorced), child sex (female; male), child race (Caucasian; multiracial; other), gestational age (weeks), exclusive breastfeeding at 6 months (yes vs no), older siblings (yes vs no), household income (<50,000; 50,000-99,999; ≥100,000; or prefer not to not say), study site (Vancouver; Edmonton; Winnipeg; Toronto), energy intake at 3 years (kcal/day), season of 3-year study visit (Spring; Summer; Winter; Autumn), attendance of childcare settings at 3 years (yes vs. no), physical activity at 5 years (hours/week), and body mass index z-score (World Health Organization reference standards) at 5 years. Abbreviations: CBCL, Child Behavior Checklist; β, beta coefficient; CI, confidence interval. A total of 585 participants had missing covariate data. * p-value < 0.05; ** p-value < 0.01.

© 2026 Kavanagh ME et al. *JAMA Network Open*.

TABLES

eTable 1. Descriptive characteristics of the original and multiple imputed datasets (n=2077) in the CHILD Cohort Study

	**Original (n = 2077)**	**Imputed (N = 2077)**
**Family Characteristics**		
Maternal Education (Post-Secondary Education vs. None)	1640 (79.0)	1674 (80.6)
Missing	40 (1.9)	0 (0.0)
Maternal Stress During Pregnancy	12.1 (6.2)	12.1 (6.1)
Missing	119 (5.8)	0 (0.0)
Maternal Marital Status		
Married	1964 (94.6)	1986 (95.6)
Single	79 (3.8)	79 (3.8)
Divorced	12 (0.6)	12 (0.6)
Missing	22 (1.0)	0 (0.0)
Maternal Energy Contribution from Ultraprocessed Food (%)	46.6 (10.6)	46.6 (10.6)
Missing	118 (5.7)	0 (0.0)
Annual Family Income		
<$50,000	172 (8.3)	187 (9.0)
$50,000-$99,999	548 (26.4)	582 (28.0)
≥$100,000	1087 (52.3)	1186 (57.1)
Prefer Not to Say	120 (5.8)	122 (5.9)
Missing	150 (7.2)	0 (0.0)
Other Siblings (Yes vs. No)	955 (46.0)	955 (46.0)
Missing	0 (0.0)	0 (0.0)
Study Site		
Edmonton	488 (23.5)	488 (23.5)
Manitoba	620 (29.9)	620 (29.9)
Toronto	449 (21.6)	449 (21.6)
Vancouver	520 (25.0)	520 (25.0)
Missing	0 (0.0)	0 (0.0)
**Birth Characteristics**		
Child Sex (Females vs Males)	985 (47.4)	985 (47.4)
Missing	0 (0.0)	0 (0.0)
Gestational age (weeks)	39.6 (1.35)	39.6 (1.35)
Missing	0 (0.0)	0 (0.0)
Child Ethnicity		

Caucasian White	1371 (66.0)	1374 (66.2)
Multiracial	480 (23.1)	481 (23.1)
Other	221 (10.6)	222 (10.7)
Missing	5 (0.2)	0 (0.0)
Exclusive Breastfeeding at 6 Months (Yes vs. No)	384 (18.7)	385 (18.5)
Missing	24 (1.2)	0 (0.0)
**Childhood Characteristics**		
**Season of Dietary Assessment at Age Three**		
Spring	489 (23.6)	489 (23.5)
Summer	555 (26.8)	557 (26.8)
Autumn	524 (25.3)	526 (25.3)
Winter	505 (24.4)	505 (24.3)
Missing	4 (0.2)	0 (0.0)
Energy Intake at Age Three (kcal/day)	1562 (498)	1562 (498)
Missing	0 (0.0)	0 (0.0)
**Energy Contributed at Age Three**		
Minimally Processed Food	37.9 (11.1)	37.9 (11.1)
Missing	0 (0.0)	0 (0.0)
Ultraprocessed Foods	45.5 (11.6)	45.5 (11.6)
Missing	0 (0.0)	0 (0.0)
Childcare Attendance at Age Three (Yes vs. No)	1152 (55.5)	1218 (58.6)
	116 (5.6)	0 (0.0)
Screen Time Age Five (hours/day)	1.29 [1.0;2.0]	1.29 [1.0;2.1]
Missing	144 (6.9)	0 (0.0)
Physical Activity at Age Five (hours/week)	2.0 [1.0, 3.0]	2.0 [1.0, 3.0]
Missing	154 (7.4)	0 (0.0)
Body Mass Index Z-score at Age Five (World Health Organization Growth Standards)	0.3 (1.0)	0.3 (1.0)
Missing	83 (4.0)	0 (0.0)

Values are frequency counts and percentages (%) for categorical variables, means and standard deviation (SD) for continuous variables.

© 2026 Kavanagh ME et al. *JAMA Network Open*.

eTable 2. Descriptive characteristics and non-response analysis of participants included in the analysis (n=2077) and participants excluded due to missing child behavioral checklist (CBCL) data (n = 327) in the CHILD Cohort Study

	**Participants included in the analysis (n = 2077)**	**Participants excluded from study due to no CBCL data at five-year visit (n = 327) ***
**Family Characteristics**		
Maternal Education (Post-Secondary Education vs. None)	1640 (79.0)	220 (66.3) †
Missing	40 (1.9)	16 (4.8)
Maternal stress during pregnancy	12.1 (6.2)	12.5 (6.5)
Missing	119 (5.8)	35 (10.5)
Maternal Marital Status		
Married	1964 (94.6)	296 (90.5) †
Single	79 (3.8)	24 (7.3) †
Divorced	12 (0.6)	3 (1.0) †
Missing	22 (1.0)	4 (1.2)
Maternal Energy Contribution from Ultra-Processed Food	46.6 (10.6)	46.3 (10.5)
Missing	118 (5.7)	35 (10.5)
Annual Family Income		
<$50,000	172 (8.3)	34 (10.2) †
$50,000-$99,999	548 (26.4)	75 (22.6) †
≥$100,000	1087 (52.3)	116 (34.9) †
Prefer Not to Say	120 (5.8)	31 (9.3) †
Missing	150 (7.2)	256 (77.1)
Other Siblings (Yes vs. No)	955 (46.0)	141 (42.5)
Missing	0 (0.0)	0 (0.0)
Study Site		
Edmonton	488 (23.5)	65 (19.6) †
Manitoba	620 (29.9)	109 (32.8) †
Toronto	449 (21.6)	90 (27.1) †
Vancouver	520 (25.0)	68 (20.5) †
Missing	0 (0.0)	0 (0.0)
**Birth Characteristics**		
Child Sex (Females vs. Males)	985 (47.4)	164 (49.4)
Missing	0 (0.0)	0 (0.0)
Child Ethnicity		
Caucasian	1371 (66.0)	200 (60.2) †
Multiracial	480 (23.1)	77 (23.2) †
Other	221 (10.6)	51 (15.4) †
Missing	5 (0.2)	4 (1.2)

© 2026 Kavanagh ME et al. *JAMA Network Open*.

Gestational age, days	39.7 [38.9; 40.6]	39.6 [38.6; 40.2]
	28 (1.3)	7 (2.1)
Exclusive Breastfeeding at 6 Months (Yes vs. No)	384 (18.5)	54 (16.3)
Missing	24 (1.2)	18 (5.4)
Childcare Attendance at Age Three (Yes vs. No)	1152 (55.5)	156 (47.7)
Missing	116 (5.6)	53 (16.2)
Daily Caloric Intake (kcal/day) at Age Three	1562.0 [1211.7; 1817.3]	1642.3 [1202.2; 1955.3] †
	0 (0.0)	0 (0.0)
Energy Contributed from NOVA Groups at Age Three		
Minimally Processed Food	37.9 (11.1)	38.5 (11.6)
Processed Culinary Ingredient	2.4 (3.2)	2.7 (3.2)
Processed Food	14.2 (5.4)	14.3 (5.9)
Ultra Processed Food	45.5 (11.6)	44.5 (11.5)
Missing	0 (0.0)	0 (0.0)
Season of Dietary Assessment		
Spring	555 (26.7)	95 (28.6)
Summer	524 (25.2)	90 (27.1)
Autumn	489 (23.5)	79 (23.8)
Winter	505 (24.3)	66 (19.9)
Missing	4 (0.2)	2 (0.6)
Child Behavior Checklist		
Internalizing	44.6 (9.1)	NA
Externalizing	39.6 (9.4)	NA
Total	41.2 (9.0)	NA
Missing	0 (0.0)	327 (100.0)
Screen time at Age Five (hours/day)	1.3 [1.0;2.0]	1.3 [1.0; 2.3]
Missing	42 (2.0)	27 (8.1)
Physical Activity at Age Five (hours/week)	2.0 [1.0, 3.0]	2.0 [1.3, 4.0]
Missing	154 (7.4)	271 (8.3)
Body Mass Index Z-score at Age Five (World Health Organization Growth Standards)	0.3 (1.0)	0.3 (1.1)
Missing	83 (4.0)	107 (32.7)

Values are frequency counts and percentages (%) for categorical variables, means and standard deviation (SD) for continuous variables. Characteristics were compared using an unpaired *t* test for normally distributed variables, Mann-Whitney test for non-normally distributed variables, and χ^2 test for categorical variables.

* A total of 327 children were excluded due to missing outcome data at 5-year visit.

† p-value < 0.05

© 2026 Kavanagh ME et al. *JAMA Network Open*.

eReferences

1. Khandpur N, Rossato S, Drouin-Chartier JP, et al. Categorising ultra-processed foods in large-scale cohort studies: evidence from the Nurses' Health Studies, the Health Professionals Follow-up Study, and the Growing Up Today Study. *J Nutr Sci.* 2021;10:e77.
2. Mendoza K, Hu FB. Are all ultra-processed foods created equal? Relevance of food processing and nutritional quality. *Eur J Epidemiol.* 2025;40(11):1289-1292.

© 2026 Kavanagh ME et al. *JAMA Network Open*.
