# Supplementary material for: Ultraprocessed Food Consumption and Behavioral Outcomes in Canadian Children
Source: JAMA Netw Open. 2026 Mar 3;9(3):e260434. doi: 10.1001/jamanetworkopen.2026.0434 (PMC12958080; doi:10.1001/jamanetworkopen.2026.0434)
Supplement: Supplement 2. — Data Sharing Statement [file jamanetwopen-e260434-s002.pdf]

# Data Sharing Statement

Kavanagh. Ultraprocessed Food Consumption and Behavioral Outcomes in Canadian Children. *JAMA Netw Open*. Published March 03, 2026.
doi:10.1001/jamanetworkopen.2026.0434

**Data**
**Data available:** No

**Additional Information**
**Explanation for why data not available:** Data described in the manuscript will be made available upon request pending approval from CHILD's Access and Publication Committee and the CHILD Study National Coordinating Centre. A list of variables available in the CHILD Cohort Study is available at https://childstudy.ca/for-researchers/study-data/. Researchers interested in collaborating on a project and accessing CHILD Cohort Study data should contact the Study's National Coordinating Centre (NCC) to discuss their needs before initiating a formal request. To contact the NCC, please email child@mcmaster.ca. More information about data access for the CHILD Cohort Study can be found at https://childstudy.ca/for-researchers/data-access/.
